# Temporal patterns of loneliness and their associations with mental health outcomes: Observations from a longitudinal study

**DOI:** 10.1192/j.eurpsy.2025.10055

**Published:** 2025-06-30

**Authors:** Błażej Misiak, Paweł Liśkiewicz, Jerzy Samochowiec

**Affiliations:** 1 Department of Psychiatry, Wroclaw Medical University, Wroclaw, Poland; 2 Department of Psychiatry, Pomeranian Medical University, Szczecin, Poland

**Keywords:** anxiety disorder, depression, paranoia, perceived social isolation, social disconnection

## Abstract

**Background:**

Loneliness is a global public health concern. Investigating loneliness in the general population offers a greater generalizability across various levels of health-related impairments, the identification of at-risk individuals, the detection of different loneliness severity levels, and broader insights into social determinants. Previous studies have shown that loneliness might be a transient or chronic experience, depending on how consistently it is reported across at least two timepoints. This study aimed to assess differential associations of chronic and transient with various domains of psychopathology.

**Methods:**

Participants were enrolled from the general population and assessed at two timepoints spanning 6–7 months. Depressive symptoms, generalized anxiety, social anxiety, and paranoid thoughts were measured using self-reports. The data were analyzed using binary logistic regressions.

**Results:**

Altogether, 3,275 participants completed both assessments with a retention rate of 64.2%. Chronic loneliness was associated with higher baseline and follow-up scores across all symptom domains. The strongest association was observed for social anxiety. Transient loneliness was not robustly associated with symptom scores. It was not significantly associated with depressive symptoms (at either of timepoints) and paranoid ideation (at baseline). The strongest association was observed for generalized anxiety. Chronic loneliness, compared to transient loneliness, was associated with significantly higher odds of social anxiety, depressive symptoms, and paranoid ideation, but not generalized anxiety.

**Conclusions:**

Both transient and chronic loneliness are associated with mental health outcomes, with the latter one showing generally stronger associations. Risk stratification and early intervention among individuals experiencing loneliness might be needed to prevent the development of more severe psychopathology.

## Introduction

The perception of social bonds has evolved significantly over time and was shaped by cultural, social, and historical contexts [1]. Pre-modern perspectives posited social isolation (currently conceptualized as an objective measure denoting that individual social bonds are lower than average) as a choiceful or even esteemed experience associated with spiritual reflection, creativity, or even philosophical inquiry [2, 3]. The term “loneliness,” understood today as a painful self-perception of social disconnection or the discrepancy between actual and desired social [4], was not commonly used or conceptualized by ancient and medieval societies [3]. The concept of loneliness, as a distressing experience, emerged more prominently in the 20th century and appeared to be influenced by societal shifts related to industrialization, urbanization, and the rise of individualism [5]. According to Bauman’s “liquid modernity” concept, these processes are largely unpredictable and coincide with the “virtualization of reality” manifesting in an ongoing digitalization of daily life and social interactions [6, 7]. The 20th century brought a growing academic interest in the psychological aspects of loneliness, with early studies that began to link loneliness with depression and anxiety [8, 9].

Currently, loneliness is perceived as a global public health concern due to its high prevalence rates and a negative impact on all aspects of health, well-being, and development [10, 11]. Experiencing loneliness has been associated with a 26% increased risk of mortality over time [12]. Taking into account the mental health perspective, it is needed to note the bidirectional associations of loneliness with symptoms across a variety of mental health outcomes, including mood and anxiety disorders [13–15], psychosis [16, 17], substance use disorders [18, 19], and problematic internet use [20]. It is also important to note that the contextual factors underlying the emergence of loneliness might show some interindividual variability [21]. For instance, factors related to social identity formation, social rejection, life transition, and digital overconnectivity might be of greater importance in younger populations [22–24]. In turn, contexts related to the loss of significant social connections, retirement, institutional care, physical illness, and related disability might play an important role in older adults [25–27].

Studying loneliness in the general population offers several advantages over focusing on clinical samples. Although clinical samples provide valuable insights into loneliness among people with poor health status, population-based studies allow for a more comprehensive understanding of loneliness as a public health concern. Studies based on the general population include a wide range of participants, providing the opportunity to apply the findings to the whole society. Given that these studies offer to study loneliness alongside a variety of demographic, social, behavioral, and environmental factors, potential risk and protective factors might be identified. Another benefit is related to the whole spectrum of loneliness severity, thereby providing an opportunity for risk stratification. At this point, it is needed to note that population-based studies hold the potential to detect at-risk individuals who show a lower severity of loneliness. Therefore, it might be foreseen that these studies hold the potential to contribute to the development of early intervention strategies applicable to primary care. Finally, existing evidence indicates that the effects of psychotherapeutic interventions on loneliness might be limited as reported effect size estimates are mostly small-to-medium [28, 29]. These observations might indicate the necessity to adopt more comprehensive approaches that also cover top-down strategies at the level of public health interventions. However, their development might require broader insights into population-wide processes.

Following the considerations about the spectrum of loneliness severity that might be covered by population-based studies, it is needed to note that previous studies have found loneliness to be either a transient or chronic experience [30–36]. Although the exact threshold duration of loneliness defining its chronic temporal pattern has not been established so far, previous studies have conceptualized chronic loneliness as its occurrence across at least two timepoints spanning between 1 and 6 years [30–36]. Investigating the differences between chronic and transient loneliness might be of importance to understand interindividual variability of underlying the mechanisms and outcomes. According to the cognitive and evolutionary model [4, 37, 38], the experience of loneliness might be adaptive as it triggers cognitions and behaviors that aim to restore social connections. However, some individuals, especially those remaining lonely over time, focus on threats related to social interactions and thus remain socially disconnected [39]. In agreement with this model, it has been observed that transient loneliness is associated with a smaller interpersonal distance, whereas chronic loneliness is related to a greater interpersonal distance [40]. A recent population-based study further demonstrated that chronic and transient loneliness show important differences in terms of underlying risk factors [41]. In this study, female gender, not being married, poor educational attainment, poor mental and physical health, being limited in activities, poor social network, and living in a culturally individualistic country are risk factors for chronic loneliness. For transient loneliness, the effects of some risk factors, including gender, physical health, level of education, and social network size, were either not significant or not robust. It has also been suggested that transient loneliness tends to occur after stressful events (e.g., retirement and loss of close social interactions), while chronic loneliness is more closely related to poor social cognition, low social support, and a lack of intimate relationships [42].

Little is known about the differential associations between chronic and transient loneliness with mental health outcomes. To date, the temporal patterns of loneliness have not been tested with respect to mental health outcomes beyond those related to depressive symptoms. It has recently been observed that both chronic and transient loneliness, assessed over the course of 1 year, are significantly associated with a higher likelihood of reporting a history of depression diagnosis and psychiatric distress [30]. However, stronger associations were found for chronic loneliness. Similar observations have been obtained in older populations [33, 36, 43, 44] and college students [45]. To bridge existing research gaps, this study aimed to assess the differential associations of chronic and transient loneliness with a variety of mental health outcomes represented by depressive symptoms, generalized anxiety, social anxiety, and paranoid thoughts in a large, general population sample. The hypothesis behind this study was that both temporal patterns are associated with poor mental health outcomes; however, these associations might be stronger for chronic loneliness.

## Methods

### Recruitment procedures

The cohort reported in this study was developed using the quota sampling method to provide sample representativeness with respect to age, gender, education, employment status, and place of residence. All assessments were carried out using self-reports implemented in an internet-based survey. Baseline data were collected between July and August 2024. The follow-up assessment took place in February 2025. At both timepoints, after the first invitation, potential participants received up to two reminders. Participants were enrolled by a research company using its own online access panel of registered and verified participants. The panel includes over 70,000 participants residing in all administrative regions of Poland. It is continuously being developed by means of regular campaigns. Individuals with underrepresented backgrounds (e.g., ethnic and social minorities) are continuously invited to register through additional campaigns initiated through trusted channels (e.g., ethnic media, cultural festivals, and local events). These campaigns are implemented by the staff trained on cultural sensitivity that approaches community leaders and organizations, and disseminates culturally appropriate contents (in terms of language, imagery, and messaging) with transparent information about research activities. In this study, panel members aged 18 years or older were eligible for participation. For completing both surveys, participants received incentives equivalent to 10 EUR. To optimize the reliability of responses, the accuracy checks were used. Specifically, respondents were excluded while violating any of the following accuracy checks: (1) short survey completion time (i.e., below 30% of the median completion time); (2) failure to pass attention checks (i.e., participants were asked to respond to items requesting them to select a specific answer); (3) inconsistent responses to repeated items; and (4) responses with random strings of characters. The study received approval from the Bioethics Committee at Wroclaw Medical University, Wroclaw, Poland (approval number: 553/2024), and all participants provided the online version of informed consent.

### Measures

#### Depressive symptoms

The Patient Health Questionnaire-9 (PHQ-9) was administered to measure depressive symptoms [46]. It is a nine-item questionnaire measuring the frequency of various depressive symptoms over the preceding 2 weeks, using a 4-point scale (0 – “not at all” to 3 – “nearly every day”). The total PHQ-9 score is between 0 and 27 (higher scores reflect a greater level of depressive symptoms). The PHQ-9 total score of ≥10 has optimal sensitivity and specificity in detecting depression and was used in this study [47]. The Cronbach’s alpha of the PHQ-9 was 0.897 in this study.

#### Generalized anxiety

Anxiety symptoms were recorded using the Generalized Anxiety Disorder-7 (GAD-7) [48]. It is a 7-item questionnaire (rated on a 4-point scale: 0 – “not at all,” 3 – “nearly every day”) that refers to generalized anxiety symptoms in the preceding 2 weeks (a 4-point scale; 0 – “not at all,” 3 – “nearly every day”). The total score ranges from 0 to 21, where higher scores indicate a greater level of anxiety symptoms. The optimal cut-off score of the GAD-7 to detect generalized anxiety disorder has been estimated at ≥ 10, and thus, it was used in our study [48]. The Cronbach’s alpha of GAD-7 was 0.944 in this study.

#### Social anxiety

The Social Interaction Anxiety Scale was used [49]. It is a 20-item questionnaire that covers various aspects of social anxiety. Respondents are instructed to rate the level at which each item is typical for them on a 5-point scale (0 – “not at all,” 4 – “extremely”). The cut-off score of ≥ 36 was used [50]. The Cronbach’s alpha was 0.947 in this study.

#### Paranoid ideation

Participants were asked to fill in the Revised Green et al. Paranoid Thoughts Scale [51]. It is based on two subscales measuring ideas of reference (part A, 8 items) and ideas of persecution (part B, 10 items) over the preceding month. Items are rated on a 5-point scale (0 – “not at all,” 4 – “totally”). Respondents were asked to assess experiences that did not appear as a consequence of substance use. For the persecution scale, the recommended cut-off to detect clinical levels of persecutory ideation was estimated at ≥ 11 [51]. This cut-off corresponds to moderately severe levels of ideas of reference (part A, ≥ 16 points). Therefore, in our study, the participants showing threshold scores reaching at least one of these cut-offs were classified as those showing paranoid ideation. In this study, the Cronbach’s alpha values for parts A and B were 0.930 and 0.964, respectively.

#### Social network size

To measure the social network size, the 6-item version of the Lubben Social Network Scale (LSNS-6) was used [52]. The LSNS-6 items record the number of family members and friends who are seen or heard at least once a month, with whom the respondent can talk about private matters, and who can be called upon for help (a 6-point scale). The total LSNS-6 score ranges from 0 to 20. Higher scores correspond with a greater social network size. The Cronbach’s alpha value of the LSNS-6 was 0.876 in this study.

#### Loneliness

To record the level of loneliness, the 11-item version of the De Jong Gierveld Loneliness Scale (DJGLS) was used [53, 54]. Each item is rated using a 5-point scale (potential responses are as follows: “yes!” “yes,” “more or less,” “no,” and “no!”). There are two DJGLS subscales: (1) measures emotional loneliness (six items) and (2)refers to social loneliness (five items). The total score is estimated by counting positive and neutral responses (“yes!” “yes,” and “more or less”) to items for emotional loneliness as well as negative and neutral responses (“no!” “no,” and “more or less”) to items developed for social loneliness. The total DJGLS score ranges between 0 and 11 (higher scores reflect a greater level of loneliness). We used the cut-off proposed by the authors of DJGLS of >2 to classify participants as those experiencing loneliness [53]. In this study, the Cronbach’s alphas for emotional and social loneliness subscales were 0.874 and 0.810, respectively.

### Data analysis

In the first step, descriptive characteristics of participants who completed assessments at both timepoints and follow-up non-completers were compared using the chi-square test (categorical variables) and t-tests (continuous variables). Next, participants who completed assessments at both timepoints were divided into three groups according to the threshold scores of the DJGLS [53], as proposed by previous studies [30, 32–36, 43, 55]. These groups were as follows: (1) individuals with a DJGLS score of <3 at both time points (i.e., participants without loneliness at neither of timepoints); (2) individuals with a DJGLS score of ≥ 3 at one timepoint (i.e., participants with transient loneliness); and (3) individuals with a DJGLS score of ≥ 3 at both timepoints (i.e., participants with chronic loneliness). Finally, binary logistic regression models were analyzed to assess differential associations of loneliness temporal patterns with symptom scores. Separate models were analyzed for baseline and follow-up symptoms taking into consideration three specific comparisons (i.e., chronic loneliness vs. no loneliness, transient loneliness vs. no loneliness, and chronic loneliness vs. transient loneliness). Covariates included age, gender, level of education, place of residence, employment status, mean monthly income, social network size, substance use in the preceding month (except for alcohol and nicotine), and psychiatric treatment history (in the preceding month). There were no missing data among individuals who completed assessments at both timepoints. Results were interpreted as significant when the *p*-value was lower than 0.05. All analyses were carried out in the SPSS software, version 28.

## Results

### Descriptive characteristics of the sample

Altogether, 10,985 individuals were approached for participation (Supplementary Figure 1). The baseline assessment was completed by 5,099 individuals (aged 44.9 ± 15.4 years, 47.7% men, 46.4% of individuals invited to participate), whereas the follow-up assessment included 3,275 individuals, resulting in a retention rate of 64.2%. Individuals who completed both assessments did not differ significantly from individuals who did not participate in the follow-up assessment with respect to sociodemographic and clinical characteristics (Table 1). Chronic loneliness was observed in 58.1% of participants, whereas transient loneliness was found in 16.2% of individuals.

**Table 1.** The general characteristics of the cohort

	Total sample(*n* = 5,099)	Completers(*n* = 3,275)	Non-completers(*n* = 1,824)	*p*
Age, years	44.9 ± 15.4	45.2 ± 15.7	44.8 ± 15.2	0.189
Gender, men	2431 (47.7)	1568 (47.9)	863 (47.3)	0.718
Education Primary Vocational Secondary Higher	94 (1.8) 411 (8.1) 2098 (41.1) 2496 (49.0)	53 (1.6) 273 (8.3) 1345 (41.1) 1604 (49.0)	41 (2.2) 138 (7.6) 753 (41.3) 892 (48.9)	0.858
Employment Unemployed Retired/disability pension Student Part-time work Full-time work	435 (8.5) 1002 (19.6) 234 (4.6) 482 (9.5) 2946 (57.8)	265 (8.1) 632 (19.3) 156 (4.8) 317 (9.7) 1905 (58.1)	170 (9.3) 370 (20.3) 78 (4.3) 165 (9.0) 1041 (57.1)	0.406
Place of residence Rural Urban, <50,000 inhabitants Urban, 50,000–150,000 inhabitants Urban, 150,000–500,000 inhabitants Urban, >500,000 inhabitants	1650 (32.4) 1166 (22.9) 816 (16.0) 714 (14.0) 753 (14.7)	1065 (32.5) 750 (22.9) 525 (16.0) 457 (14.0) 478 (14.6)	585 (32.1) 416 (22.8) 291 (16.0) 257 (14.0) 275 (15.1)	0.991
Monthly income <750 USD 750–1500 USD 1,500–2,500 USD 2,500–3,750 USD >3,750 USD Refused to answer	1082 (21.1) 2485 (48.7) 722 (14.2) 133 (2.6) 44 (0.9) 633 (12.4)	695 (21.2) 1621 (49.6) 468 (14.3) 80 (2.4) 31 (0.9) 380 (11.6)	387 (21.2) 864 (47.4) 254 (13.9) 53 (2.9) 13 (0.7) 253 (13.9)	0.170
Social network size, baseline	15.9 ± 6.0	16.0 ± 6.0	15.8 ± 5.8	0.222
Social network size, follow-up	–	15.7 ± 5.9	–	–
Psychiatric treatment Lifetime Preceding month	1108 (21.7) 539 (10.6)	705 (21.5) 327 (10.0)	403 (22.1) 512 (28.1)	0.638 0.068
Substance use, preceding month^a^	353 (6.9)	213 (6.5)	140 (7.7)	0.353
Depression, baseline	1362 (26.7)	860 (26.3)	502 (27.5)	0.329
Depression, follow-up	–	823 (25.1)	–	–
Generalized anxiety, baseline	1084 (21.3)	683 (20.9)	401 (22.0)	0.345
Generalized anxiety, follow-up	–	664 (20.3)	–	–
Social anxiety, baseline	1890 (37.1)	1206 (36.8)	684 (37.5)	0.632
Social anxiety, follow-up	–	1219 (37.2)	–	–
Paranoid ideation, baseline	1618 (31.7)	1017 (31.1)	601 (32.9)	0.163
Paranoid ideation, follow-up	–	1018 (31.1)	–	–
Loneliness Chronic Transient None	– – –	1904 (58.1) 532 (16.2) 839 (25.6)	– – –	–

*Note*: Data are reported as mean ± SD or *n* (%).

a Except for nicotine and alcohol.

### Baseline symptoms and temporal patterns of loneliness

**Figure 1.**
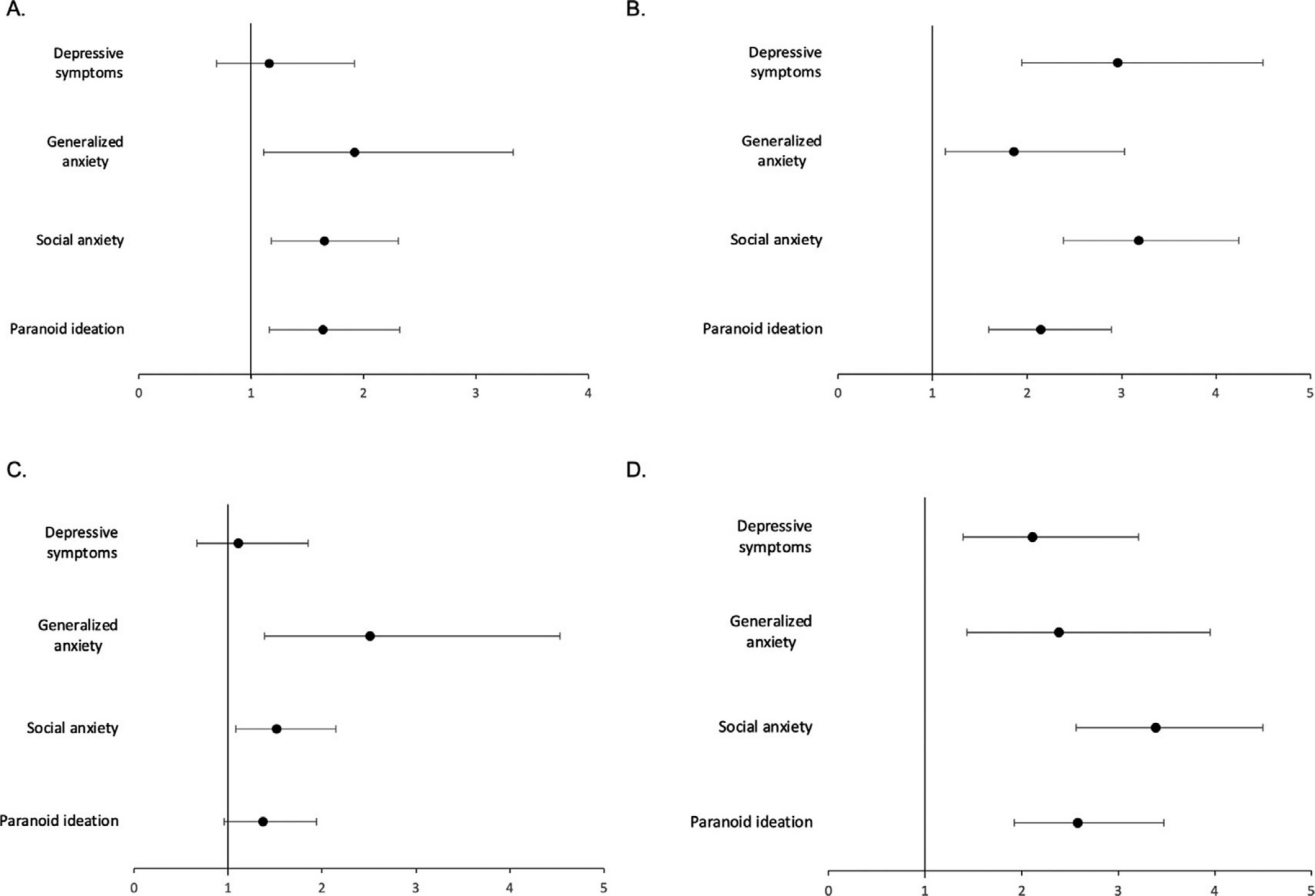
Temporal patterns of loneliness and their associations with mental health outcomes. The associations with baseline symptoms are shown in Figure 1A (transient loneliness vs. no loneliness) and Figure 1B (chronic loneliness vs. no loneliness), while the associations with follow-up symptoms are visualized in Figure 1C (transient loneliness vs. no loneliness) and Figure 1D (chronic loneliness vs. no loneliness). Results are adjusted for age, gender, education, place of residence, employment status, monthly income, social network size, substance use (in the preceding month), and psychiatric treatment history (in the preceding month).

Transient loneliness was associated with significantly higher odds of baseline generalized anxiety, social anxiety, and paranoid ideation, but not with depressive symptoms. This observation remained significant after adjustment for covariates (Table 2, Figure 1A). The highest effect size estimate was found for generalized anxiety (unadjusted analysis: OR = 1.94, 95%CI: 1.16–3.27, *p* = 0.012; adjusted analysis: OR = 1.92, 95%CI: 1.11–3.33, *p* = 0.020). Among all tested covariates, only a lower social network size at baseline was significantly associated with transient loneliness.

**Table 2.** The associations of baseline mental health measures with temporal patterns of loneliness

Model	Independent variable	Analysis					
		Unadjusted			Adjusted		
		OR	95%CI	p	OR	95%CI	p
Transient loneliness vs. no loneliness	Depressive symptoms	1.20	0.74–1.94	0.455	1.16	0.69–1.92	0.578
	Generalized anxiety	1.94	1.16–3.27	**0.012**	1.92	1.11–3.33	**0.020**
	Social anxiety	1.87	1.36–2.56	**<0.001**	1.65	1.18–2.31	**0.004**
	Paranoid ideation	1.62	1.16–2.24	**0.004**	1.64	1.16–2.32	**0.005**
	Age	–	–	–	0.99	0.98–1.00	0.134
	Gender (women)	–	–	–	1.26	0.97–1.62	0.084
	Education	–	–	–	1.18	0.98–1.43	0.081
	Urbanicity	–	–	–	0.96	0.87–1.04	0.311
	Unemployment^a^	–	–	–	1.01	0.76–1.34	0.967
	Income	–	–	–	1.04	0.88–1.23	0.626
	Psychiatric treatment^b^	–	–	–	1.54	0.82–2.90	0.176
	Substance use^c^	–	–	–	1.87	0.90–3.89	0.094
	Social network size	–	–	–	0.89	0.87–0.92	**<0.001**
Chronic loneliness vs. no loneliness	Depressive symptoms	3.39	2.35–4.90	**<0.001**	2.96	1.94–4.50	**<0.001**
	Generalized anxiety	1.85	1.20–2.86	**0.006**	1.86	1.14–3.03	**0.013**
	Social anxiety	4.29	3.35–5.50	**<0.001**	3.18	2.38–4.24	**<0.001**
	Paranoid ideation	2.17	1.67–2.82	**<0.001**	2.14	1.59–2.89	**<0.001**
	Age	–	–	–	0.99	0.98–1.05	0.326
	Gender (women)	–	–	–	1.39	1.10–1.76	**0.005**
	Education	–	–	–	1.26	1.07–1.49	**0.007**
	Urbanicity	–	–	–	0.95	0.88–1.03	0.252
	Unemployment^a^	–	–	–	0.93	0.72–1.20	0.569
	Income	–	–	–	0.97	0.83–1.13	0.661
	Psychiatric treatment^b^	–	–	–	1.02	0.62–1.66	0.950
	Substance use^c^	–	–	–	2.27	1.22–4.22	**0.009**
	Social network size	–	–	–	0.80	0.78–0.82	**<0.001**

*Note*: Significant associations (*p* < 0.05) are bolded.

a The lack of any employment and student status.

b The prior-month history.

c The prior-month history; nicotine and alcohol use were not assessed.

Chronic loneliness was associated with significantly higher baseline levels of all symptom scores before and after adjustment for covariates (Table 2, Figure 1B). The highest effect size estimate was observed for social anxiety (unadjusted analysis: OR = 4.29, 95%CI: 3.35–5.50, *p* < 0.001; adjusted analysis: OR = 3.18, 95%CI: 3.18, 95%CI: 2.38–4.24, *p* < 0.001). Chronic loneliness was also significantly associated with female gender, higher levels of education, higher odds of substance use, and a lower social network size.

### Follow-up symptoms and temporal patterns of loneliness

Transient loneliness was associated with significantly higher odds of follow-up generalized anxiety and social anxiety. This observation was significant before and after adjustment for covariates (Table 3, Figure 1C). The highest effect size estimate was found for generalized anxiety (unadjusted analysis: OR = 2.57, 95%CI: 1.46–4.53, *p* = 0.001; adjusted analysis: OR = 2.51, 95%CI: 1.39–4.53, *p* = 0.002). Among all tested covariates, only a lower social network size at the follow-up was significantly associated with transient loneliness.

**Table 3.** The associations of follow-up mental health measures with temporal patterns of loneliness

Model	Independent variable	Analysis					
		Unadjusted			Adjusted		
		OR	95%CI	p	OR	95%CI	p
Transient loneliness vs. no loneliness	Depressive symptoms	1.12	0.69–1.82	0.656	1.11	0.67–1.85	0.690
	Generalized anxiety	2.57	1.46–4.53	**0.001**	2.51	1.39–4.53	**0.002**
	Social anxiety	1.77	1.28–2.45	**<0.001**	1.52	1.08–2.15	**0.017**
	Paranoid ideation	1.30	0.93–1.81	0.126	1.37	0.96–1.94	0.081
	Age	–	–	–	0.99	0.98–1.00	0.147
	Gender (women)	–	–	–	1.26	0.97–1.63	0.080
	Education	–	–	–	1.21	1.00–1.46	0.050
	Urbanicity	–	–	–	0.97	0.89–1.06	0.485
	Unemployment^a^	–	–	–	0.99	0.74–1.31	0.929
	Income	–	–	–	1.06	0.90–1.24	0.517
	Psychiatric treatment^b^	–	–	–	1.39	0.74–2.60	0.303
	Substance use^c^	–	–	–	2.03	0.98–4.23	0.058
	Social network size	–	–	–	0.89	0.86–0.91	**<0.001**
Chronic loneliness vs. no loneliness	Depressive symptoms	2.26	1.57–3.24	**<0.001**	2.11	1.39–3.21	**<0.001**
	Generalized anxiety	2.92	1.84–4.62	**<0.001**	2.38	1.43–3.95	**<0.001**
	Social anxiety	5.00	3.89–6.41	**<0.001**	3.39	2.56–4.50	**<0.001**
	Paranoid ideation	2.34	1.81–3.03	**<0.001**	2.58	1.92–3.47	**<0.001**
	Age	–	–	–	0.99	0.98–1.01	0.639
	Gender (women)	–	–	–	1.36	1.08–1.72	**0.009**
	Education	–	–	–	1.25	1.05–1.48	**0.010**
	Urbanicity	–	–	–	0.97	0.90–1.05	0.475
	Unemployment^a^	–	–	–	0.90	0.70–1.16	0.422
	Income	–	–	–	0.97	0.83–1.13	0.690
	Psychiatric treatment^b^	–	–	–	0.97	0.60–1.58	0.915
	Substance use^c^	–	–	–	2.42	1.31–4.47	**0.005**
	Social network size	–	–	–	0.79	0.77–0.81	**<0.001**

*Note*: Significant associations (*p* < 0.05) are bolded.

a The lack of any employment and student status.

b The prior-month history.

c The prior-month history; nicotine and alcohol use were not assessed.

Chronic loneliness was associated with significantly higher odds of all symptoms before and after adjustment for covariates (Table 3, Figure 1D). The highest effect size estimate was found for social anxiety (unadjusted analysis: OR = 4.29, 95%CI: 3.35–5.50, *p* < 0.001; adjusted analysis: OR = 3.18, 95%CI: 2.38–4.24, *p* < 0.001). Chronic loneliness was also significantly associated with female gender, higher levels of education, higher odds of substance use, and a lower social network size.

### Differences between temporal patterns of loneliness in their associations with mental health outcomes

**Figure 2.**
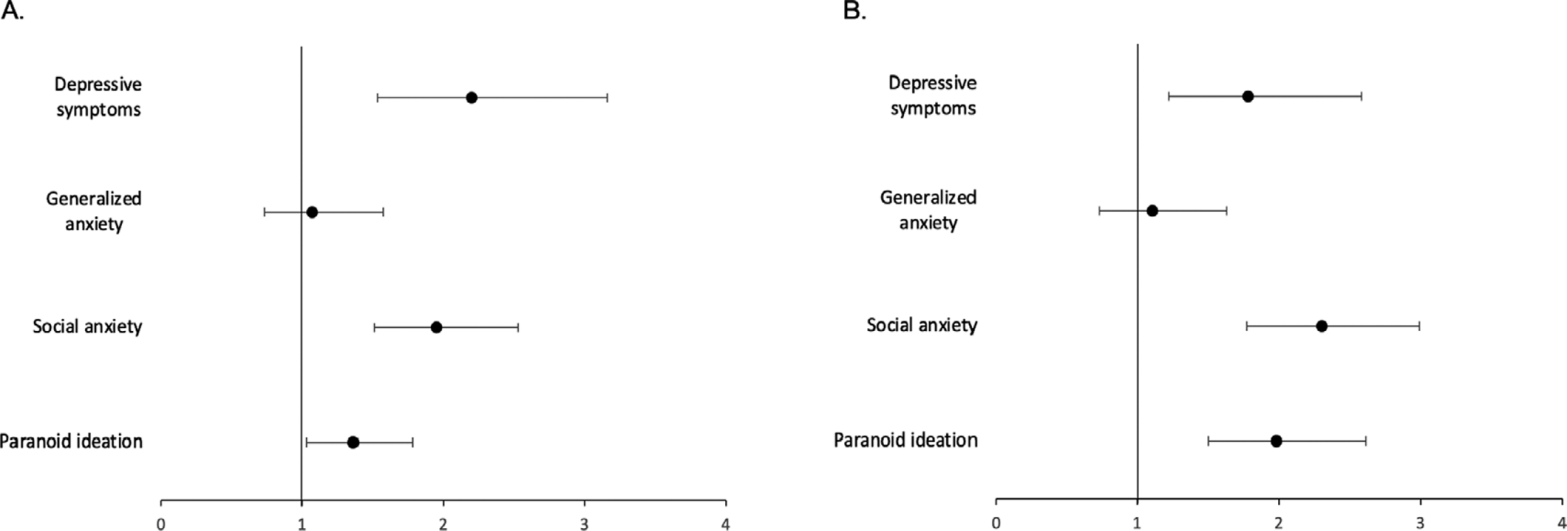
Differences between temporal patterns of loneliness in their associations with baseline (A) and follow-up (B) symptoms. Chronic loneliness is a reference category. Results are adjusted for age, gender, education, place of residence, employment status, monthly income, social network size, substance use (in the preceding month), and psychiatric treatment history (in the preceding month).

Chronic loneliness, compared to transient loneliness, showed stronger associations with baseline and follow-up symptoms of depression, social anxiety, and paranoid thoughts, but not generalized anxiety (Table 4, Figure 2). These differences remained significant after adjustment for covariates. With respect to baseline symptoms, the highest effect size estimate was found for depressive symptoms (unadjusted analysis: OR = 2.46, 95%CI: 1.74–3.49, *p* < 0.001; adjusted analysis: OR = 2.20, 95%CI: 1.53–3.16, *p* < 0.001). In turn, for follow-up symptoms, the highest effect size estimate was observed for social anxiety (unadjusted analysis: OR = 2.68, 95%CI: 2.10–3.44, *p* < 0.001; adjusted analysis: OR: 2.30, 95%CI: 1.77–2.99, *p* < 0.001).

**Table 4.** Differences between chronic and transient loneliness with respect to mental health measures

Model	Independent variable	Analysis					
		Unadjusted			Adjusted		
		OR	95%CI	p	OR	95%CI	p
Baseline measures of mental health	Depressive symptoms	2.46	1.74–3.49	**<0.001**	2.20	1.53–3.16	**<0.001**
	Generalized anxiety	1.05	0.72–1.52	0.808	1.07	0.73–1.57	0.731
	Social anxiety	2.29	1.80–2.91	**<0.001**	1.95	1.51–2.53	**<0.001**
	Paranoid ideation	1.33	1.03–1.72	**0.030**	1.36	1.03–1.78	**0.028**
	Age	–	–	–	1.00	0.99–1.01	0.718
	Gender (women)	–	–	–	1.21	0.96–1.53	0.111
	Education	–	–	–	1.06	0.89–1.25	0.520
	Urbanicity	–	–	–	0.98	0.90–1.06	0.566
	Unemployment^a^	–	–	–	0.95	0.73–1.22	0.675
	Income	–	–	–	0.94	0.82–1.09	0.440
	Psychiatric treatment^b^	–	–	–	0.74	0.46–1.19	0.215
	Substance use^c^	–	–	–	0.85	0.51–1.42	0.536
	Social network size	–	–	–	0.89	0.87–0.91	**<0.001**
Follow-up measures of mental health	Depressive symptoms	1.82	1.28–2.59	**<0.001**	1.78	1.22–2.58	**0.003**
	Generalized anxiety	1.16	0.79–1.70	0.444	1.10	0.73–1.63	0.657
	Social anxiety	2.68	2.10–3.44	**<0.001**	2.30	1.77–2.99	**<0.001**
	Paranoid ideation	1.78	1.37–2.32	**<0.001**	1.98	1.50–2.61	**<0.001**
	Age	–	–	–	1.00	0.99–1.01	0.727
	Gender (women)	–	–	–	1.18	0.93–1.50	0.166
	Education	–	–	–	1.06	0.89–1.26	0.495
	Urbanicity	–	–	–	0.98	0.91–1.07	0.668
	Unemployment^a^	–	–	–	0.95	0.74–1.23	0.695
	Income	–	–	–	0.95	0.82–1.10	0.481
	Psychiatric treatment^b^	–	–	–	0.73	0.46–1.17	0.195
	Substance use^c^	–	–	–	0.83	0.49–1.39	0.474
	Social network size	–	–	–	0.88	0.86–0.90	<0.001

*Note*: Chronic loneliness was included as reference. Significant associations (*p* < 0.05) are bolded.

a The lack of any employment and student status.

b The prior-month history.

c The prior-month history; nicotine and alcohol use were not assessed.

## Discussion

### Main findings

Findings from this study indicate that chronic loneliness, compared to its transient temporal pattern, shows stronger and robust associations with the symptoms of social anxiety, generalized anxiety, depression, and paranoia that are likely to be observed in a short-term perspective. In case of chronic loneliness, these associations are likely to be bidirectional, with the largest effect size estimates observed for social anxiety. In case of transient loneliness, associations with psychopathological symptoms might be more complex. First, it is important to note that our study did not demonstrate any significant associations of transient loneliness with depressive symptoms. Second, transient loneliness was significantly associated with higher baseline but not follow-up odds of paranoid ideation. Third, the strongest effect driven by transient loneliness was found for generalized anxiety, and this effect was comparable to that found for chronic loneliness.

In general, the findings about quantitative differences in the strength of associations with symptom domains between chronic and transient loneliness are in agreement with our expectations and observations from previous studies measuring the level of depressive symptoms [30, 33, 36, 43–45]. Several processes might explain the observed associations between loneliness and mental health outcomes. There is evidence that individuals experiencing loneliness tend to show a number of social information-processing biases that might increase the risk of developing mental disorders. These include attention for threat bias, negative and hostile intent attributions, increased rejection sensitivity, negative evaluation of self and others, endorsement of less promotion- and more prevention-oriented goals, and low perceived self-efficacy [39]. These cognitive biases might be bidirectionally associated with depression [56], generalized anxiety disorder [57], social anxiety disorder [58], and psychosis [59].

It is also important to discuss some discrepancies between our findings and previous observations. Indeed, our findings, to some extent, contradict the evolutionary model of loneliness, positing its transient temporal pattern as an adaptive experience that signals the need to reconnect with others and indicating a lack of unfavorable associations with mental health. However, observations from previous studies also do not support these considerations. For instance, a longitudinal study of adolescents with an observation period of 12 years identified three trajectories of loneliness, i.e., “stable low,” “high decreasing,” and “low increasing” [60]. Both “high decreasing” and “low increasing” temporal patterns were associated with elevated risk of depression. In addition, the “low increasing” pattern was associated with a higher risk of anxiety. Another study, based on a 14-year observation period of older adults, revealed two frequency patterns of loneliness (i.e., moderate and high frequency), and both were associated with increased risk of depression [61]. Also, the study, which spanned 1 year and included two waves of data, demonstrated that both chronic and transient loneliness are related to a higher risk of depression [30]. Notably, this study did not demonstrate any significant associations of transient loneliness with depressive symptoms while observing significant associations with social and generalized anxiety as well as follow-up paranoid ideation. This difference might originate from the fact that our study, as opposed to previous studies, analyzed the associations with depressive symptoms while accounting for co-occurring psychopathology.

### Methodological considerations

To the best of our knowledge, this study has various strengths related to broad insights into symptom domains associated with loneliness, a relatively large sample size, and a longitudinal design. However, the findings need to be interpreted in light of some limitations.

Recruitment procedures were implemented among the online panel users. While this approach provided representativeness of the sample in terms of sociodemographic characteristics, the risk of selection bias should be considered. Indeed, the study was performed among internet users and initiated during the summer period, when many people spend holidays. Moreover, we did not use any measures of problematic internet use that has been associated with loneliness [20]. Following these considerations, it cannot be excluded that individuals with problematic internet use and those with higher levels of loneliness were overrepresented in the present cohort. Another limitation is that we did not record the information about the social or ethnic minority status. Also, the response and retention rates (46.4 and 64.2%, respectively) were relatively low. However, a recent meta-analysis estimated the mean response rate across online surveys at 44.1% [62]. Moreover, the response rates are not always directly related to the validity of findings [63]. For instance, it has been found that studies with low response rates, even those with a response rate of 20%, are able to provide more accurate results than those with a response rate of 60–70% [64].

Another limitation is related to a lack of general consensus on how loneliness should be operationalized as a categorical construct. Therefore, the prevalence rates of loneliness show relatively high variability across previous studies, which is further influenced by the use of specific questionnaires. In general, studies using single-item measures tend to report lower prevalence rates, and some authors argue that simple measures may not thoroughly capture the construct of loneliness [65, 66]. A recent meta-analysis estimated the prevalence of loneliness at 55.4% in community-dwelling older adults while pooling the studies using DJGLS [65]. For studies based on single-item questions, the prevalence rate was found to be 21.2%. Single-item measures often use direct indicators of loneliness (e.g., “I feel lonely” and “How often do you feel lonely?”). Indirect indicators, represented by the DJGLS that was used in this study, are not based on the reference to loneliness. Therefore, they do not evoke negative stereotypes and limit socially desirable responses [66, 67]. Indeed, individuals experiencing loneliness are likely to hold self-stigmatizing perceptions that might be defined as “the shame for being lonely and inclination to conceal loneliness” [68]. In this regard, the use of single-item and direct indicators might lead to underreporting of loneliness. Findings from our study may also provide limited insights into the temporal patterns of loneliness due to a low number of assessments over time and a relatively short observation period. However, we believe that approaching shorter observation periods may also be important, as they inform us about the early consequences of loneliness and the processes that directly precede its occurrence. Considering our approach to data analysis, it is needed to note that while we controlled for the effects of various sociodemographic characteristics, we did not assess whether these variables moderate the associations of loneliness with mental health outcomes.

It should also be noted that while the general population provides opportunities for studying loneliness as a public health concern, taking into consideration the heterogeneity of social determinants, this approach limits the potential to translate findings over individuals with mental disorders. At this point, it is also important to highlight that the measures of mental health outcomes were based on screening questionnaires, and a comprehensive clinical validation was not performed. Finally, it should be noted that the study does enable to draw conclusions about causality due to the observational design.

### Directions for future studies

The most important direction for future studies is related to the need of a consensus-driven operationalization of loneliness temporal patterns. In this regard, two research directions need to be discussed. First, it should be considered to develop questionnaires recording the duration of loneliness. Second, it might be needed to perform intensive longitudinal studies that record the experience of loneliness and mental health characteristics over multiple timepoints in order to find the threshold duration defining temporal patterns of loneliness. Another important direction is related to the need for a better understanding of factors moderating the associations of loneliness with mental health in order to define populations with the highest vulnerability. For instance, it has consistently been shown that the prevalence of loneliness is the highest in young adulthood (up to 30 years of age), decreases in middle adulthood and early old age, and then increases during the oldest old age [69–71]. To date, it has been suggested that contextual factors underlying loneliness in various age groups might differ [22–27]. However, as shown by a recent meta-analysis, interventions targeting loneliness in various age groups might show comparable efficacy [29].

Finally, with an ongoing digitalization and frequent involvement in remote social interactions, it might be needed to better understand on how various problematic online behaviors are interrelated with loneliness. A recent meta-analysis demonstrated that problematic internet use is bidirectionally associated with loneliness [20]. This association was found to be stronger in samples from Eastern countries, with more men and young adults, and those studied over recent years. On the one hand, problematic internet use might be perceived as a strategy to cope with social disconnection (either loneliness or social isolation) and boredom [72–74]. On the other hand, problematic internet use may increase the level of loneliness through the mechanisms related to social displacement, a lack of adequate sensory cues, and bodily feedback [75, 76]. However, it is needed to point out that problematic internet use is now believed to serve as the spectrum of various problematic online behaviors that show differential associations with mental health outcomes [77]. Addressing their differential associations with loneliness is warranted. Also, the need to explore on how digital advancements might be reframed to tackle loneliness should be highlighted. For instance, there is some evidence that enhancing video communication between care home residents and family members might be feasible in supporting person-centered care, social interactions, and well-being [78].

### Clinical implications

Although this study was carried out in the general population, some implications for public health and clinical practice might be formulated. The findings imply the necessity to consider early intervention and risk stratification, taking into consideration the temporal pattern of loneliness. It should also be considered which interventions need to be prioritized among individuals showing specific temporal patterns of loneliness. According to the cognitive-evolutionary model, transient loneliness might motivate individuals to seek social interactions [4, 37, 38]. A recent study revealed that while transient loneliness is associated with a smaller preferred interpersonal distance, chronic loneliness was found to be linked with a greater preferred interpersonal distance [40]. In this regard, it might be concluded that specific therapeutic interventions targeting transient loneliness, especially those related to motivating social involvement, may not be necessary. In this group, awareness of the transition to chronic loneliness, improvement of social skills, maintaining close relationships, community engagement, and clinical assessment should be considered [79]. In turn, the initiation of therapeutic interventions might be the priority for individuals with chronic loneliness. To date, a variety of approaches targeting chronic loneliness have been developed. A recent meta-analysis revealed that priority should be given to reminiscence interventions, interventions promoting social identity formation, and cognitive-behavioral therapy [29]. Another meta-analysis revealed the greatest effect size for cognitive interventions, but also a lack of benefits related to interventions that enhance social skills [80].

## Conclusions

Taken together, the findings indicate that chronic and transient loneliness show qualitative and quantitative differences in their associations with psychopathological symptoms that might already be observed in a short-term perspective. Chronic loneliness shows robust and bidirectional associations across various domains of psychopathology, covering depressive symptoms, social anxiety, generalized anxiety, and paranoid ideation. In turn, transient loneliness shows bidirectional associations with generalized and social anxiety. These observations hold some implications referring to the public health and clinical perspectives by indicating the rationale to detect loneliness in its early development. However, additional studies approaching longer observation periods and thorough clinical assessments are also needed to provide broader insights into the mechanisms, consequences, and temporal patterns of loneliness.

## Supporting information

10.1192/j.eurpsy.2025.10055.sm001 Misiak et al. supplementary material Misiak et al. supplementary material

## Data Availability

Due to sensitivity reasons, the data supporting this study are available upon reasonable request sent to the corresponding author.

## Supplementary material

The supplementary material for this article can be found at http://doi.org/10.1192/j.eurpsy.2025.10055.
