# Supplementary figures and images for: Temporal patterns of loneliness and their associations with mental health outcomes: Observations from a longitudinal study
Source: Eur Psychiatry. 2025 Jun 30;68(1):e94. doi: 10.1192/j.eurpsy.2025.10055 (PMC12303777; doi:10.1192/j.eurpsy.2025.10055)

**Supplementary Appendix**

**Figure 1.** The flow diagram of participants.


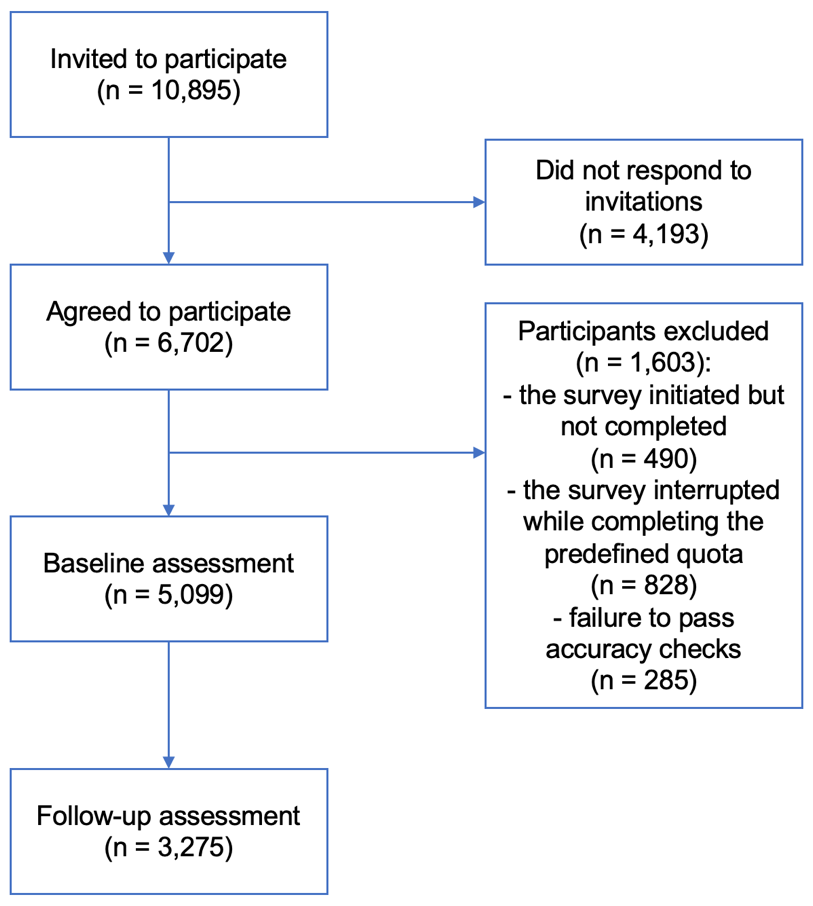

Supplement: Misiak et al. supplementary material [file S0924933825100552sup001.docx]
